# *Mycobacterium tuberculosis* Induction of Heme Oxygenase-1 Expression Is Dependent on Oxidative Stress and Reflects Treatment Outcomes

**DOI:** 10.3389/fimmu.2017.00542

**Published:** 2017-05-12

**Authors:** Neesha Rockwood, Diego L. Costa, Eduardo P. Amaral, Elsa Du Bruyn, Andre Kubler, Leonardo Gil-Santana, Kiyoshi F. Fukutani, Charles A. Scanga, JoAnne L. Flynn, Sharon H. Jackson, Katalin A. Wilkinson, William R. Bishai, Alan Sher, Robert J. Wilkinson, Bruno B. Andrade

**Affiliations:** ^1^Wellcome Centre for Infectious Disease Research in Africa, Institute of Infectious Disease and Molecular Medicine, University of Cape Town, Cape Town, South Africa; ^2^Department of Medicine, Imperial College, London, UK; ^3^Immunobiology Section, Laboratory of Parasitic Diseases, National Institutes of Allergy and Infectious Diseases, National Institutes of Health, Bethesda, MD, USA; ^4^Infectious Diseases and Immunity, Imperial College, London, UK; ^5^Center for Tuberculosis Research, Johns Hopkins University School of Medicine, Baltimore, MD, USA; ^6^Instituto Gonçalo Moniz, Fundação Oswaldo Cruz, Salvador, Brazil; ^7^Multinational Organization Network Sponsoring Translational and Epidemiological Research (MONSTER) Initiative, Fundação José Silveira, Salvador, Brazil; ^8^Curso de Medicina, Faculdade de Tecnologia e Ciências, Salvador, Brazil; ^9^Department of Microbiology and Molecular Genetics, University of Pittsburgh School of Medicine, Pittsburgh, PA, USA; ^10^Division of Intramural Research, National Institute on Minority Health and Health Disparities, National Institutes of Health, Bethesda, MD, USA; ^11^The Francis Crick Institute, London, UK; ^12^Division of Infectious Diseases, Department of Medicine, Vanderbilt University School of Medicine, Nashville, TN, USA; ^13^Escola Bahiana de Medicina e Saúde Pública, Salvador, Brazil

**Keywords:** tuberculosis, HIV, heme oxygenase-1, biomarker, oxidative stress

## Abstract

The antioxidant enzyme heme oxygenase-1 (HO-1) is implicated in the pathogenesis of tuberculosis (TB) and has been proposed as a biomarker of active disease. Nevertheless, the mechanisms by which *Mycobacterium tuberculosis* (*Mtb*) induces HO-1 as well as how its expression is affected by HIV-1 coinfection and successful antitubercular therapy (ATT) are poorly understood. We found that HO-1 expression is markedly increased in rabbits, mice, and non-human primates during experimental *Mtb* infection and gradually decreased during ATT. In addition, we examined circulating concentrations of HO-1 in a cohort of 130 HIV-1 coinfected and uninfected pulmonary TB patients undergoing ATT to investigate changes in expression of this biomarker in relation to HIV-1 status, radiological disease severity, and treatment outcome. We found that plasma levels of HO-1 were elevated in untreated HIV-1 coinfected TB patients and correlated positively with HIV-1 viral load and negatively with CD4^+^ T cell count. In both HIV-1 coinfected and *Mtb* monoinfected patients, HO-1 levels were substantially reduced during successful TB treatment but not in those who experienced treatment failure or subsequently relapsed. To further delineate the molecular mechanisms involved in induction of HO-1 by *Mtb*, we performed a series of *in vitro* experiments using mouse and human macrophages. We found that *Mtb*-induced HO-1 expression requires NADPH oxidase-dependent reactive oxygen species production induced by the early-secreted antigen ESAT-6, which in turn triggers nuclear translocation of the transcription factor NRF-2. These observations provide further insight into the utility of HO-1 as a biomarker of both disease and successful therapy in TB monoinfected and HIV-TB coinfected patients and reveal a previously undocumented pathway linking expression of the enzyme with oxidative stress.

## Introduction

Tuberculosis (TB) is an airborne disease caused by infection with *Mycobacterium tuberculosis* (*Mtb*) bacilli. It remains a major global health problem as approximately 1.7 billion people are estimated to be infected (1). In 2015, it was estimated that 1.4 million people died from TB alone with an additional 0.4 million deaths as a result of TB-HIV coinfection, making TB 1 of the 10 top causes of morbidity and death worldwide (2).

The pathogenesis of TB involves activation of myeloid cells, which promotes the generation of free radicals (3, 4). However, due to the broad diversity of clinical manifestations, TB infection outcomes can vary from asymptomatic infection [latent TB infection (LTBI)] to severe progressive lung disease with extensive destruction of pulmonary tissue (5–7). Both clinical and experimental animal studies in TB have described excessive oxidative cellular and tissue damage leading to lipid peroxidation and cell death (8–11). The intense inflammatory environment of active TB is hallmarked by accumulation of reactive oxygen species (ROS), which are largely driven by mitochondria *via* NADPH oxidase (12).

Heme oxygenase-1 (HO-1) is a potent antioxidant enzyme, which is induced in response to cellular stress (10, 13, 14). In several experimental systems, HO-1 expression is triggered in response to heme accumulation (15), exposure to toxic arsenic compounds (16, 17), hypoxia (18), starvation (19), as well as toll-like receptor (TLR) and cytokine-mediated cellular activation (20–22). The enzyme catalyzes the degradation of heme molecules into free iron, biliverdin, and carbon monoxide (CO), the latter product exhibiting anti-inflammatory and cytoprotective effects (14). The protective versus detrimental roles of host HO-1 during TB infection are still controversial and incompletely understood. Mice genetically deficient in HO-1 are more susceptible to mycobacterial infection (23). However, these knockout animals also exhibit profound hematopoietic abnormalities (24, 25) that could provide an alternative explanation of the phenotype observed in *Mtb* infection.

There is additional contrary evidence that HO-1 expression may be associated with rather than suppress TB infection and pathology. Indeed, in studies on South Indian and Brazilian patients, circulating levels of HO-1 were increased in both adult and pediatric TB patients compared to uninfected persons and patients with LTBI (26–28). In addition, TB patients exhibiting clinical and radiographic signs of more severe illness displayed significantly higher HO-1 levels in plasma than those who had milder TB disease (10, 27). The concept that HO-1 may actually promote TB infection is supported by experiments in which growth of *Mtb* in macrophages was suppressed by addition of a pharmacological inhibitor of host HO-1 enzymatic activity (29). The same drug when given to *Mtb*-infected mice induced a substantial decrease in pulmonary mycobacterial loads when administered in conjunction with anti-TB antibiotic therapy and accelerated bacterial clearance (30). Together, these results argue that in the context of *Mtb* infection, overexpression of HO-1 may be detrimental rather than beneficial.

The molecular mechanisms underlying HO-1 induction by *Mtb* are only partially understood. As mentioned earlier, infection of murine and human macrophages with *Mtb* induces robust production of HO-1 (10) and mycobacterial infection of mice also triggers pulmonary expression of the enzyme (23, 30–32). We have previously shown that HO-1 induction in human macrophages requires live replicating *Mtb* and the expression of ESAT-6 (6 kDa early secretory antigenic target) (10). Nevertheless, the downstream intermediates through which this *Mtb* virulence factor triggers HO-1 transcription have not been defined.

In this study, we have extended our analysis of HO-1 as a biomarker of active TB infection and further delineated the mechanisms through which *Mtb* induces HO-1 production in infected cells. First, we analyzed *in vivo* HO-1 expression in experimental *Mtb* infection of rabbits, mice, and non-human primates (NHP) and confirmed in these different animal models that HO-1 levels are associated with active infection and disease and diminish upon antitubercular therapy (ATT). In addition, using plasma samples from a large cohort of clinically well-characterized TB and HIV-1-TB patients in South Africa, we analyzed for the first time the influence of concomitant HIV-1 infection on TB-induced HO-1 expression and correlated enzyme levels with treatment outcome. Finally, in *in vitro* studies using infected monocyte-derived human macrophages and bone marrow-derived murine macrophages, we demonstrated that the mechanism by which *Mtb* ESAT-6 stimulates HO-1 expression involves the induction of NADPH-derived ROS production leading to activation and translocation of the transcription factor nuclear factor erythroid-derived 2-like 2 (NRF-2). Taken together, these findings further delineate the pathway through which *Mtb* infection triggers HO-1 expression while demonstrating the potential use of the enzyme as biomarker for disease and treatment outcome in the complex setting of a population where TB and HIV-1 infections coexist.

## Results

### *Mtb* Infection Drives HO-1 Expression at the Disease Site and Systemically in Three Experimental Models of Pulmonary TB

**Figure 1:**
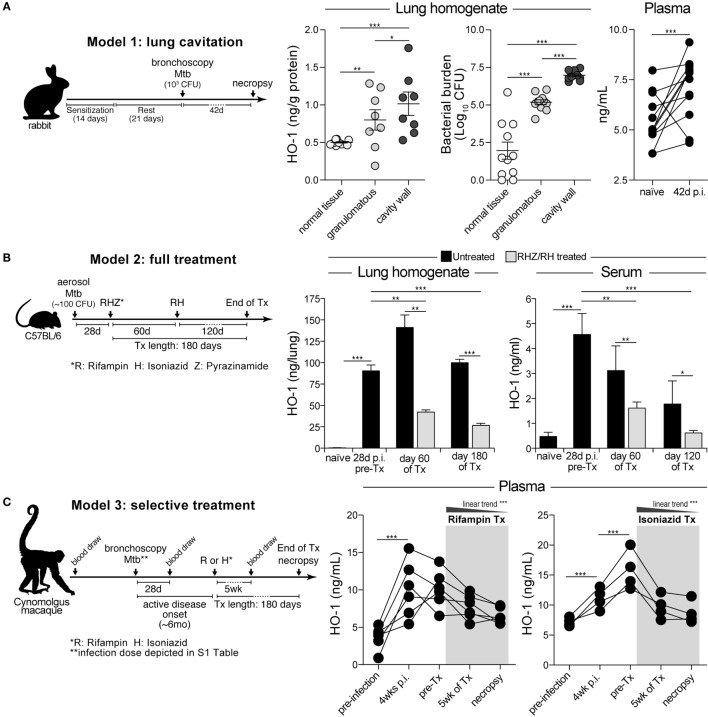
**Experimental *Mycobacterium tuberculosis* (*Mtb*) infection *in vivo* induces heme oxygenase-1 (HO-1) expression in lungs and blood, which is reversed during antitubercular therapy**. **(A)** HO-1 protein expression was quantitatively assessed in lung homogenates and in plasma samples from rabbits bronchoscopically infected with *Mtb* by ELISA. **(B)** HO-1 levels in lung homogenates and serum samples were measured in a tuberculosis treatment model using mice infected with a low dose of *Mtb*. Graph shows representative data from different experiments using 4 mice in each time point per experimental group (total animal number = 8–12 mice per group). **(C)** Prospective changes in HO-1 concentrations in plasma were evaluated in non-human primates experimentally infected with *Mtb* and treated with rifampin or isoniazid. Data were analyzed using the Mann–Whitney *U* test (two unmatched groups), the Kruskal–Wallis test with Dunn’s multiple comparisons or linear trend *ad hoc* tests (more than two groups) or the Wilcoxon matched-pairs test (*p < 0.05, **p < 0.01, ***p < 0.0001).

We have previously shown that circulating levels of HO-1 are increased in pulmonary TB patients compared to uninfected individuals and those with LTBI in both adults and children (27, 28). In these studies, patients were recruited at the time of disease diagnosis and for this reason we did not have data on their history of TB infection. In order to temporally examine the induction of HO-1 following *Mtb* infection, we quantified its expression in three distinct animal models. Using a recently published model of lung cavitation in rabbits (33, 34), we observed that HO-1 protein expression increased in lung areas with granulomatous lesions compared to those with normal tissue (Figure 1A). HO-1 expression was further augmented in the cavity walls (Figure 1A). Notably, the level of HO-1 expression reflected bacterial burden in the different lung areas in infected animals (Figure 1A). Pulmonary infection of rabbits with *Mtb* was also associated with a significant increase in HO-1 plasma levels (Figure 1A). These results indicate that circulating levels of HO-1 reflect the induction of HO-1 protein expression at the infection site. In addition, they show that HO-1 expression correlates with the bacterial burden in different areas of the infected lung.

We next examined HO-1 expression in lungs and in serum in a mouse model of pulmonary TB (35), before and after a chemotherapy regimen that closely mimics that used in humans. In agreement with a recent study from our group (30), we found that HO-1 expression in both lungs and serum significantly increased following aerosol infection with *Mtb* (Figure 1B). Initiation of ATT reduced HO-1 induction, with concentrations substantially falling in the serum of mice receiving the antimicrobials compared to untreated animals (Figure 1B). Interestingly, although greatly reduced, significant levels of HO-1 expression remained relative to the levels displayed by uninfected control mice perhaps due to the presence of persistent bacteria.

Experimental infection of NHP has been described to induce a pulmonary disease that more accurately resembles human TB than that occurring in murine and rabbit models (36). We therefore measured HO-1 levels in plasma of *cynomolgus* macaques bronchoscopically infected with *Mtb* and treated with rifampin or isoniazid when active TB disease was established (37). In this NHP model, HO-1 levels significantly increased as early as 4 weeks postinfection and remained elevated following development of active TB (Figure 1C). Treatment with either rifampin or isoniazid was associated with gradual decreases in HO-1 concentrations (Figure 1C). At the end of therapy, treated animals exhibited HO-1 levels indistinguishable from those detected at preinfection. These findings in multiple animal models strongly support the association of HO-1 expression levels with TB infection and disease as well as the potential utility of the enzyme as biomarker of successful anti-TB therapy.

### Plasma Levels of HO-1 Distinguish Successful from Failed Treatment or Relapse in Pulmonary TB Patients from South Africa

**Figure 2:**
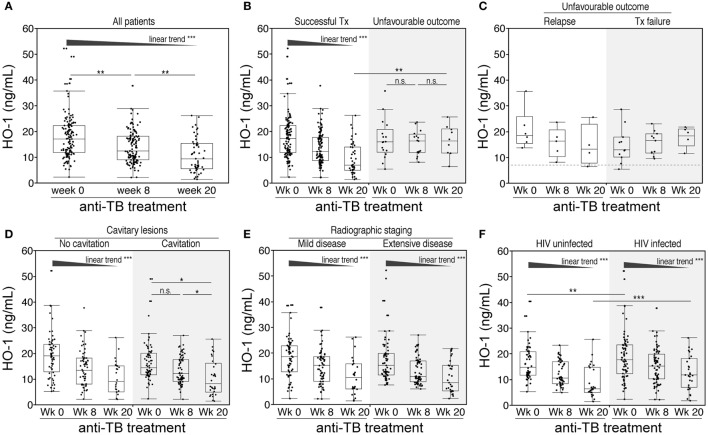
**Changes in plasma concentrations of heme oxygenase-1 (HO-1) in pulmonary tuberculosis (TB) patients undergoing antitubercular treatment**. **(A)** Plasma levels of HO-1 were prospectively assessed in samples from 130 patients with pulmonary TB before and at the indicated time points after anti-TB treatment initiation. **(B,C)** HO-1 levels in patients with successful outcome and in those who had unfavorable outcomes (treatment failure or relapse). HO-1 levels at different time points upon anti-TB treatment were also stratified by the presence of cavitary lesions **(D)**, extensive disease on X-ray at pretreatment **(E)**, or HIV infection status **(F)**. Data were analyzed using Friedman matched pairs with Dunn’s multiple comparisons test as well as non-parametric linear trend *post hoc* test.

Our previous clinical data suggested that measuring HO-1 expression in patients undergoing ATT could potentially discriminate different treatment outcomes (27). To more rigorously test this hypothesis, we examined HO-1 concentrations in plasma from a well-characterized cohort of 130 South African patients with active pulmonary TB from whom serial samples were collected. HO-1 concentrations gradually diminished during treatment (Figure 2A). Notably, while levels of the enzyme consistently decreased in individuals who were successfully treated, they did not significantly change in patients who exhibited unfavorable outcomes (e.g., treatment failure or TB relapse at the end of therapy) (Figure 2B). Plasma concentrations of HO-1 were similar between patients who exhibited TB relapse or treatment failure during the study follow-up irrespective of the time point (Figure 2C), although the small number of patients in these subgroups of unfavorable outcomes preclude a definite conclusion. Similarly, prior to therapy, HO-1 levels were indistinguishable between patients who were successfully treated and those who were not (Figure 2B). Nevertheless, by week 20 of therapy, patients with unfavorable outcomes exhibited HO-1 levels substantially higher than those successfully treated (Figure 2B). These findings demonstrate that pretreatment levels of HO-1 cannot be used to predict the outcome of TB therapy, but the extent of HO-1 expression measured at the end of therapy does reflect outcome. To quantitatively assess the accuracy of HO-1 levels at week 20 of therapy in discriminating successful treatment from failure or relapse, we performed receiver operator characteristics (ROC) curve analysis. We confirmed that HO-1 levels at the end of anti-TB treatment exhibited high accuracy in classifying treatment outcomes (area under the curve: 0.81, sensitivity: 90%, specificity 67.4%, *p* = 0.003; Figure S1 in Supplementary Material).

Additional analysis revealed that within the entire study population, HO-1 plasma concentrations did not differ between patients presenting with cavitary or non-cavitary disease (Figure 2D). Patients with non-cavitary disease exhibited a gradual decrease in HO-1 levels upon treatment initiation, while those with cavitary disease displayed significant changes in HO-1 concentrations only at the end of therapy. Prior to therapy, circulating concentrations of HO-1 were not different between patients classified as having mild or extensive radiographic disease (Figure 2E). Moreover, treatment-induced reductions in HO-1 levels were independent of the radiographic extent (Figure 2E).

### HIV Disease Progression Affects HO-1 Expression in TB Patients

**Figure 3:**
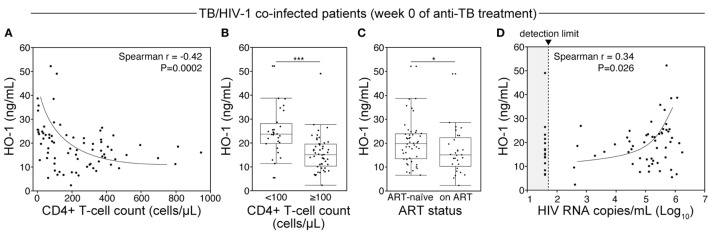
**HIV-1 coinfection and degree of immunosuppression relates to heme oxygenase-1 (HO-1) concentrations in plasma from tuberculosis (TB) patients prior to antitubercular treatment**. **(A)** Spearman correlation analysis of HO-1 levels in plasma by CD4^+^ T-cell counts in HIV-infected patients before initiation of antibubercular therapy. **(B)** HIV-infected patients were further classified based on CD4^+^ T-cell counts and HO-1 levels. **(C)** HO-1 levels were also compared between antiretroviral therapy (ART)-naïve patients and those who were on ART before TB treatment. In panels **(B,C)**, data were compared using the Mann–Whitney *U* test. **(D)** Spearman correlation analysis of HO-1 levels in plasma with HIV viral load at pretreatment in HIV-infected patients.

In our patient cohort, 75 (57.7%) patients were coinfected with HIV-1 (Table 1). Of those, 48 (64%) were antiretroviral therapy (ART) naïve. Of note, HO-1 concentrations in plasma were significantly higher in HIV-coinfected than in HIV-1-uninfected patients before treatment initiation (Figure 2F). Although HO-1 levels substantially decreased upon treatment initiation irrespective of HIV status, HIV-coinfected patients had higher values than those of uninfected subjects at the end of TB therapy (Figure 2F). These results suggest that HIV-1 coinfection may influence the regulation of HO-1 expression in TB patients. We next tested whether HIV-1-driven immunosuppression is associated with increased HO-1 expression in TB patients. In TB/HIV coinfected patients, plasma levels of HO-1 negatively correlated with CD4^+^ T-cell counts (Figure 3A) before anti-TB treatment. Individuals who were highly immunosuppressed (CD4^+^ T-cell count <100 cells/μL) exhibited increased HO-1 concentrations compared to those who were not (Figure 3B). Patients who were on ART at week 0 of ATT had lower HO-1 levels than those who were ART-naïve (Figure 3C). In addition, HO-1 levels positively correlated with HIV-1 viral load (Figure 3D). These findings suggest that HIV-1 coinfection causes further increases in HO-1 expression in TB patients.

**Table 1:** **Characteristics of study participants**.

Characteristic	All	HIV-1 infected	HIV-1 uninfected	*p*-Value
*N*	**130**	**75**	**55**	
**Male—no. (%)**	73 (56.1)	32 (42.7)	41 (74.5)	0.0003
**Median age—years (IQR)**	35.1 (30–44)	35.1 (10.5–42.1)	35.4 (27.5–51)	0.994
**Median BMI—kg/m^2^ (IQR)**	21 (19–23)	22 (20–24)	20 (19–23)	0.035
**Comorbidities—no. (%)**				
Diabetes mellitus	9 (6.9)	4 (5.3)	5 (9.1)	0.489
Smoking history	60 (46.1)	29 (38.6)	31 (56.4)	0.048
**AFB smear—no. (%)**				0.015
Negative/scanty	46 (35.4)	34 (45.4)	12 (21.8)	
1+	24 (18.5)	15 (20)	9 (16.4)	
2+	28 (21.5)	13 (17.3)	15 (27.3)	
3+	32 (24.6)	13 (17.3)	19 (34.5)	
**Extensive radiological disease—no. (%)**	91 (70.0)	41 (54.6)	50 (90.9)	<0.0001
**Cavitation—no. (%)**	67 (51.5)	33 (44)	34 (61.8)	0.049
**Culture conversion at week 8—no. (%)**	75 (57.7)	49 (65.3)	26 (47.3)	0.070
**Death—no. (%)**	5 (3.8)	5 (6.7)	0	0.058

*Frequency data between HIV-infected and -uninfected active TB patients were analyzed using the Fisher’s exact test or the Pearson’s chi-square test. Continuous variables were compared using the Mann–Whitney U test. p-Values were adjusted for multiple comparisons*.

### Induction of HO-1 in Human and Mouse Macrophages Infected with *Mtb* Requires NADPH-Derived ROS Production

**Figure 4:**
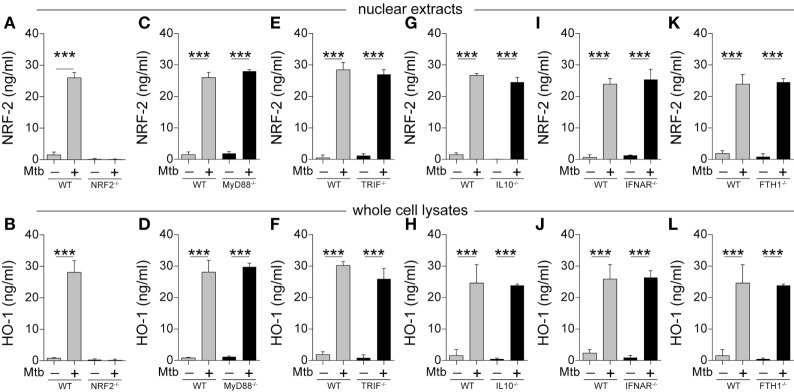
***Mycobacterium tuberculosis* (*Mtb*) induction of heme oxygenase-1 (HO-1) protein expression in mouse macrophages is independent of multiple signaling pathways linked to innate immunity in tuberculosis**. **(A–L)** Bone marrow-derived macrophages from mice genetically lacking expression of the indicated genes or their respective wild-type (WT) controls were infected with H37Rv *Mtb* (multiplicity of infection: 3) for 24 h. Protein expression of nuclear factor erythroid-derived 2-like 2 (NRF-2) and HO-1 was quantified in nuclear extracts and whole cells lysates, respectively, by ELISA. Bars and lines represent mean and SEM, respectively. Data are from at least three independent experiments using triplicate biological samples. Data were compared using the Wilcoxon matched-pairs test (*p < 0.05, **p < 0.01, ***p < 0.0001). MyD88, myeloid differentiation primary response gene 88; TRIF, TIR domain-containing adapter-inducing interferon-β; IL-10, interleukin-10; IFNAR, interferon alpha and beta receptor subunit 1; FTH1, ferritin heavy chain 1.

The results presented above demonstrate that HO-1 is induced upon *Mtb* infection in different experimental models *in vivo* as well as in patients with active disease. To further delineate the molecular mechanisms to induce HO-1 in the context of *Mtb* infection, we employed an *in vitro* system using mouse and human macrophages infected with *Mtb* H37Rv strain (10). We chose macrophages because this cell type is known to be an important source of HO-1 in several disease models including human and murine mycobacterial infections (29). In other HO-1 induction systems, expression of the enzyme is thought to require translocation to the nucleus of the transcription factor known as nuclear factor erythroid-derived 2-like 2 (NRF-2) (14). We found that this is also true for HO-1 induction by *Mtb* infection as bone marrow-derived macrophages (BMDMs) from mice genetically lacking NRF-2 were unable to produce HO-1 following bacterial exposure (Figures 4A,B). In our *in vitro* system, the results obtained with HO-1 protein quantification reflected the degree of *HMOX1* messenger RNA (mRNA) expression (Figure S2 in Supplementary Material).

Several signaling pathways have been described to be associated with HO-1 expression, including classical pattern-recognition pathways (38), interleukin-10 (IL-10) (39), and type I interferon (40). Remarkably, induction of both nuclear NRF-2 translocation and HO-1 protein expression was not affected in *Mtb*-infected BMDM from mice lacking MyD88, TRIF, IL-10, interferon alpha and beta receptor subunit 1 (IFNAR), or ferritin heavy chain (FTH1) (Figures 4C–L). These observations reveal that HO-1 induction driven by *Mtb* infection of macrophages involves a mechanism that is independent of numerous signaling pathways involved in molecular recognition of the pathogen and innate immune responses classically linked to TB pathogenesis.

**Figure 5:**
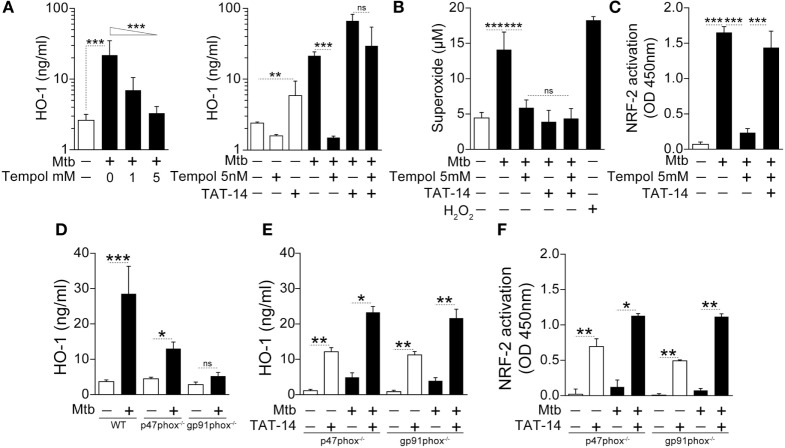
**NADPH-derived reactive oxygen species is required for induction of heme oxygenase-1 (HO-1) in macrophages infected with *Mycobacterium tuberculosis* (*Mtb*)**. **(A)** Human monocyte-derived macrophages were infected with *Mtb* H37Rv [multiplicity of infection (MOI): 3] in the presence or absence of an antioxidant (Tempol) for 24 h and HO-1 levels were measured in whole cell extracts by ELISA. Right panel shows HO-1 levels in infected macrophages treated with Tempol in the presence of a TAT-conjugated NRF-2 sequence peptide that interacts with the Keap-1/NRF-2 complex (TAT-14, 50 µM). **(B)** Generation of superoxide anions was quantified in cell supernatants of cultures shown in panel **(A)**. H_2_O_2_ was used as positive control. **(C)** Activation of the HO-1 transcription factor NRF-2 in nuclear extracts was quantitatively assessed 12 h after infection using a colorimetric DNA-binding ELISA kit. **(D)** Bone marrow-differentiated macrophages were prepared from wild type (WT), p47phox^−/−^, or gp91phox^−/−^ (deficient in two distinct subunits of the NADPH oxidase system) mice and infected *in vitro* with *Mtb* (MOI: 3) for 24 h. HO-1 levels were then assessed in whole cell extracts by ELISA. **(E,F)** HO-1 levels in cell extracts as well as activation of NRF-2 in nuclear extracts were quantified in macrophage cultures in the presence of TAT-14 (50 µM). Data are from at least three experiments using cells from a total of up to six healthy donors. **(D–F)** Three independent experiments were performed, with samples run in triplicates. Data from different biological groups were analyzed using the Kruskal–Wallis test, with the Dunn’s multiple-comparison test, whereas matched analyses were performed using the Wilcoxon matched-pairs test (*p < 0.05, **p < 0.01, ***p < 0.001). ns, non-significant; NRF-2, nuclear factor erythroid-derived 2-like 2.

We have recently described that patients with active pulmonary TB exhibit elevated circulating levels of oxidation products and lipid peroxidation compared with individuals with LTBI or uninfected controls, as well as in human macrophages infected with *Mtb in vitro*, demonstrating an important role for oxidative stress in this disease (11). In this study, we asked whether the oxidative stress triggered by *Mtb* infection might play a role in driving HO-1 expression. Indeed, treatment of *Mtb*-infected human monocyte-derived macrophage cultures with Tempol, a potent antioxidant, caused a substantial reduction in HO-1 protein expression (Figure 5A, left panel).

In unstimulated conditions, NRF-2 has been described to interact with the protein Kelch-like ECH-associated protein 1 (Keap-1) in the cytosol of macrophages and other cell types. This interaction induces proteasome-mediated degradation of the former molecule. Upon stimulation, ROS is thought to interfere with the NRF-2/Keap-1 interaction, releasing the transcription factor, which then translocates to the nucleus to induce HO-1 (41, 42). To test if the ROS requirement for HO-1 induction in our *in vitro* system involves a similar pathway, we incubated cultures of infected human monocyte-derived macrophages treated with Tempol in the presence of TAT-14, a peptide comprising a TAT-conjugated NRF-2 sequence known to interfere with the NRF-2/Keap-1 interaction (43). Notably, HO-1 induction was restored when TAT-14 was added to the cultures of infected cells treated with Tempol (Figure 5A, right panel), without altering ROS production (Figure 5B). The restoration of HO-1 induction upon *Mtb* infection was reflected by increased activation of NRF-2 in nuclear extracts (Figure 5C). These findings demonstrate that ROS-mediated interference of NRF-2/Keap-1 interaction is a major pathway involved in the induction of HO-1 by *Mtb* in murine macrophages.

The NADPH oxidase system is a major source of ROS in both physiological and pathological conditions (44). To test if NADPH-derived ROS plays a role in *Mtb*-driven HO-1 induction, we infected BMDM from mice deficient in either one of the two main NADPH-oxidase subunits (p47Phox and gp91Phox), which lack fully functional NADPH-oxidase activity (45, 46). Importantly, *Mtb*-induced HO-1 expression was markedly diminished in the genetically deficient mice compared with the wild-type (WT) animals (Figure 5D). In these experiments, the p47Phox^−/−^ mice and their respective WT controls were from C57BL/6NTac genetic background, whereas the gp91Phox^−/−^ animals were from the C57BL/6J background. The latter genetic background has been associated with a spontaneous mutation that results in mitochondrial redox abnormalities (47), which could have influenced the HO-1 production *in vitro*. However, the pattern of HO-1 production in the experimental conditions was very similar to that observed in mice from the C57BL/6NTac genetic background. In addition, as observed with Tempol-treated human macrophages (Figure 5A), TAT-14 addition restored both HO-1 expression and NRF-2 activation (Figures 5E,F, respectively) to the NADPH oxidase-deficient macrophage cultures from both genetic backgrounds. These results support the hypothesis that *Mtb* drives HO-1 expression in infected macrophages by inducing NADPH-derived ROS, which inhibits NRF2/Keap1 interaction.

### *Mtb*-Associated Oxidative Stress and HO-1 Production Are Dependent on ESAT-6 Expression

**Figure 6:**
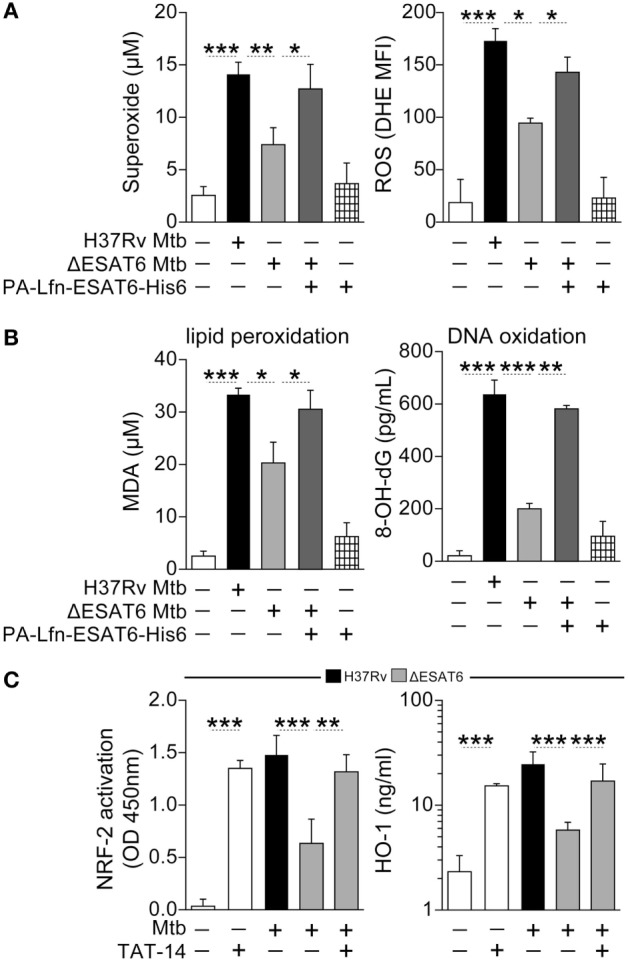
**ESAT6-mediated heme oxygenase-1 (HO-1) induction in *Mycobacterium tuberculosis* (*Mtb*)-infected macrophages is associated with reactive oxygen species (ROS)-dependent release of NRF-2 to the nucleus**. **(A)** Human monocyte-differentiated macrophages were infected with *Mtb* H37Rv or an H37Rv mutant strain lacking ESAT-6 expression (both at multiplicity of infection: 3) for 24 h. Cells infected with the ESAT-6-deficient *Mtb* strain were treated with 20 µg/mL of the fusion protein Lfn-ESAT-6 with the anthrax-protective antigen cytosolic delivery system. Generation of superoxide anions was quantified in cell supernatants using a colorimetric assay, while intracellular ROS production was assessed by flow cytometry. Results are plotted as histograms where the mean fluorescence intensity (MFI) was compared between the experimental groups. **(B)** Lipid peroxidation and oxidative DNA damage were assessed as described in Section “Materials and Methods.” **(C)** Activation of NRF-2 in nuclear extracts (12 h postinfection) and of HO-1 levels in whole cell lysates (24 h postinfection) were determined in cultures of infected macrophages in the presence or absence of a TAT-conjugated NRF-2 sequence peptide that interacts with the Keap-1/NRF-2 complex (TAT-14, 50 µM). Data are from at least three independent experiments using cells from a total of up to six healthy donors. Data were compared using the Wilcoxon matched-pairs test (*p < 0.05, **p < 0.01, ***p < 0.0001). MDA, malondialdehyde; NRF-2, nuclear factor erythroid-derived 2-like 2; 8-OH-dG, 8-hydroxy-2′-deoxyguanosine.

Many pathological effects observed in cells infected with *Mtb* are related to bacterial production of the virulence factor ESAT-6, a protein that is secreted into the host cell cytosol (48). We have previously reported that *Mtb* lacking ESAT-6 failed to induce robust HO-1 expression in both human and murine macrophages (10). Experiments were next performed to determine whether the role of ESAT-6 in HO-1 induction involves the oxidative stress-dependent pathway described above. We found that infection of human macrophages with an H37Rv mutant lacking ESAT-6 was associated with a decreased extracellular and intracellular ROS response (Figure 6A). This reduction in ROS production was linked to reduced concentrations of malondialdehyde (MDA), a marker of lipid peroxidation, and of 8-hydroxy-2′-deoxyguanosine (8-OH-dG), a readout of oxidative DNA damage (Figure 6B). We next delivered recombinant ESAT-6 protein fused with the N-terminal fragment of the lethal factor of *Bacillus anthracis* (10, 49) into the cytosol of macrophages infected with ESAT-6-deficient *Mtb*. Notably, delivery of ESAT-6 into macrophages restored ROS production (Figure 6A) and was further associated with augmented lipid peroxidation and oxidative DNA damage (Figure 6B), at levels comparable to that induced by the control WT H37Rv strain. As expected, the decreased HO-1 expression observed in macrophages infected with ESAT-6-deficient *Mtb* was associated with reduced NRF-2 activation in nuclear extracts (Figure 6C), and these defects were corrected when macrophage cultures were treated with TAT-14 (Figure 6C). Together, these findings support a link between ESAT-6 expression and ROS-induced activation of NRF-2 in the triggering of HO-1 production by *Mtb* in human macrophages.

Previous studies in both murine BMDMs and RAW264.7 cells found that exposure to soluble recombinant ESAT-6 protein triggers HO-1 expression *via* induction of the microRNA 155 (miR-155) (50). We therefore tested the participation of miR-155 in our *in vitro* system by assessing NRF-2 nuclear translocation and HO-1 production in miR-155KO murine macrophages (Figure S3 in Supplementary Material). Our experiments revealed that *Mtb*-infected BMDMs from miR-155KO mice failed to induce HO-1 production while exhibiting NRF-2 quantification in nuclear extracts similar to that observed in WT cells. Such diminished HO-1 production detected in cultures from miR-155KO cells was not reverted by treatment with TAT-14 (Figure S3 in Supplementary Material). These findings suggest that miR-155 is critical to induce HO-1 production in *Mtb*-infected macrophages by a distinct pathway that does not involve interference with NRF-2/Keap-1 interaction.

## Discussion

Heme oxygenase-1 production is induced during the course of several infectious diseases as well as inflammatory disorders and may play beneficial or detrimental role for the host depending on the stimulus and context (29, 30, 40, 51–57). *Mtb* infection, in particular, induces a robust production of the enzyme detected in plasma (27, 31, 32) suggesting its potential use as a biomarker for TB. Indeed, our previous studies on a cohort of South Indian HIV-negative TB patients revealed a correlation between HO-1 levels and active disease and demonstrated a reduction in the enzyme following successful antibiotic treatment (27). In this study, we have confirmed the association of circulating levels of HO-1 with *Mtb* infection in three different animal models and in the case of the rabbit correlated enzyme expression with pulmonary pathology. Moreover, we were able to replicate in mice and NHP the reduction in HO-1 levels observed in patients following chemotherapy.

To further examine the utility of HO-1 as an accurate biomarker of active *Mtb* infection in humans and to assess the impact of HIV coinfection on enzyme expression, we performed a prospective analysis of HO-1 levels in a South African cohort of TB and HIV-TB patients prior to and at an early time point (8 weeks) as well as at 20 weeks post-ATT. In agreement with our previous findings, a major (approximately threefold) decrease in HO-1 levels was observed in all successfully treated patients after 20 weeks of chemotherapy. More importantly, decreased enzyme levels were also evident at 8 weeks posttreatment, but only in those patients lacking cavitary lesions who went on to clear their infections. These findings suggest that measurement of HO-1 levels could be a useful correlate of successful therapy at the conclusion of conventional treatment, but is unlikely to be a reliable early predictor of treatment outcome. Whether those individuals with lowered HO-1 levels at early time points would still clear their infections if therapy was withdrawn is unclear and was not tested in this study.

Approximately 58% of the TB patients in our cohort were HIV-1 coinfected allowing us to evaluate the influence of HIV coinfection on HO-1 expression. Although it is unclear from previous studies whether HIV infection results in elevated plasma HO-1 levels, the virus has been reported to trigger downmodulation of HO-1 expression in brain tissue as well as in infected monocyte-derived macrophages (58, 59). Our results show that HIV/*Mtb* coinfected patients display higher HO-1 levels, which inversely correlated with CD4^+^ T-cell counts and positively correlated with viral load, indicating that HIV coinfection quantitatively enhances HO-1 expression in TB patients. Alternatively, the increased HO-1 found in HIV/TB patients may merely reflect the increase in enzyme levels resulting from the expected defective control of *Mtb* in these individuals (60, 61), although such an effect was not evident in the sputum smears performed in the cohort studied here (Table 1). Moreover, as described above for *Mtb* monoinfected patients, successful anti-TB treatment also resulted in reduced plasma HO-1 levels in HIV coinfected individuals. Although these observations suggest that *Mtb* is the force driving HO-1 expression in HIV coinfected patients, further studies involving individuals with HIV monoinfection and comparing the effects of antiretroviral treatment versus anti-TB therapy in coinfected subjects are needed to test this hypothesis. Another hypothesis to explain heightened HO-1 levels in HIV/TB coinfected patients is that HIV induces pyroptosis in CD4^+^ T-cells (62), subsequently leading to massive inflammation and release of cytosolic content. This would potentially explain why HIV/TB coinfected patients still display increased HO-1 levels at the end of anti-TB treatment as well as the observed reductions in serum HO-1 in patients on ART compared to ART-naïve individuals.

The mechanisms through which *Mtb* infection induces HO-1 expression *in vivo* or for that matter in infected cells are not completely understood. In the case of cultured macrophages, we and others have demonstrated a role for the *Mtb* virulence factor ESAT-6 in HO-1 induction. While our previous experiments with primary human macrophages indicated a requirement for live infection with ESAT-6 expressing bacteria and cytosolic delivery of the protein (10), Kumar et al. studying both murine BMDMs and RAW264.7 cells found that exposure to soluble recombinant ESAT-6 protein alone was sufficient to trigger HO-1 expression (50). Analyzing the mechanism responsible for HO-1 induction, the latter authors identified a major role for the micro RNA miR-155 and Bach-1 in the transcriptional regulation of HO-1. In other studies, soluble ESAT-6 has been shown to stimulate miR-155 through a TLR2, MyD88-dependent pathway (63).

The data shown in this report demonstrate a role for ROS in the ESAT-6-dependent induction of HO-1 by *Mtb*. This pathway appears to be independent of TLR, MyD88, as well as TRIF signaling and thus apparently distinct from the miR-155 pathway described by Kumar et al. (50). A link between oxidative stress and HO-1 production has been documented previously in other systems (64–66), and we and others have shown that *Mtb* infection is a potent stimulus of ROS production (11, 67). Importantly, we show in this study that ESAT-6-dependent stimulation of ROS promotes HO-1 production by inducing the release and nuclear translocation of the transcription factor NRF-2, which normally exists as a cytosolic complex with Keap-1 (41, 42). We speculate that the activation of NRF-2 by this pathway is responsible for the transcription of HO-1 induced by *Mtb* in macrophages. Keap-1 has previously been identified as a sensor of oxidative stress (68) and been linked to HO-1 expression through the NRF-2 pathway studied here (41, 42, 69). Our findings are the first to formally establish the participation of this mechanism in the induction of HO-1 by *Mtb*. In future experiments, it will be important to identify the pathway by which intracellular ESAT-6 is recognized and triggers ROS production in infected cells. In addition, further studies are needed to define the relationship of the ESAT-6/ROS/NRF-2/HO-1 pathway described here with the previously reported ESAT-6-driven miR-155-dependent mechanism that de-represses Bach-1-mediated suppression of HO-1 transcription. Conceivably, both mechanisms could coregulate expression of the enzyme in different contexts. Indeed, our experiments demonstrating that *Mtb*-infected BMDM from miR-155 KO mice fail to induce HO-1 production by a mechanism dependent on NRF-2 nuclear translocation reinforce the hypothesis that there are likely other pathways by which *Mtb* triggers HO-1 expression in macrophages.

While the data presented here add new information on the expression of HO-1 in TB infection and disease as well as the mechanisms underlying the induction of this important biomarker, the precise physiological role played by the enzyme in the immunopathogenesis of *Mtb* infection remains unclear. The biological mechanisms underlying HO-1 expression in plasma of patients is not clearly understood. HO-1 is described as a cytosolic enzyme expressed in many cell types and tissues. Our results from plasma measurements of this enzyme led us to hypothesize that in pulmonary TB patients, the increased HO-1 observed in plasma derives from injured tissues, a concept that would explain the strong association of HO-1 with bacterial burden (27). Furthermore, as discussed earlier, previous results from our group and others have shown that pharmacological inhibition of HO-1 activity can reduce *Mtb* loads *in vitro* in infected human macrophages (29) as well as *in vivo* in an experimental murine model of TB and result in accelerated bacterial clearance when used in conjunction with antibiotics (30). Nevertheless, there is contrary data to suggest that the primary role of HO-1 induction is host protection (23) consistent with the known antioxidant and cytoprotective functions of the enzyme. Further research is clearly necessary to resolve this controversy. Regardless, continued dissection of the mechanisms underlying HO-1 induction in *Mtb* infection will be important in revealing potential targets for either suppressing or enhancing this response as an approach to therapeutic intervention and for further understanding the precise parameters that HO-1 reflects when used as a biomarker for TB disease and treatment.

## Materials and Methods

### Patient Population and Clinical Procedures

Patients with GeneXpert *Mtb*/RIF-confirmed rifampin-susceptible pulmonary TB were recruited at the Ubuntu HIV/TB Clinic, Site B, Khayelitsha, South Africa, as part of a prospective cohort study. Detailed sociodemographic data, past TB treatment history, and comorbidity data were collected. At the study baseline, HIV testing (Abbott Architect HIV Ag/Ab Combo test), CD4^+^ T lymphocyte count, and HIV-1 viral load quantification were performed. Extensive disease on chest X-ray was performed by experts and noted as either involvement of >1 lung or involvement of ≥1 out of 3 (upper, mid, or lower) zones per lung. Antitubercular drugs were provided in a 4-drug fixed-dose combination supplied by the National Tuberculosis Control Programme [Rifafour e-275 (Sanofi-Aventis) or Ritib (Aspen, South Africa)]. Each tablet contained rifampin at 150 mg, isoniazid at 75 mg, pyrazinamide at 400 mg, and ethambutol at 275 mg. Weight band-based dosing was used in line with WHO guidelines (70). Patients weighing 38–55, >55–70, and >70 kg were given doses of 3, 4, and 5 tablets, respectively. Anti-TB drugs were administered 7 days/week, along with 25 mg pyridoxine. Clinical care remained the responsibility of the Site B TB clinic. Unfavorable treatment outcomes were defined as failure or relapse. Failure was any TB patient who was culture positive at 5 months or more after starting treatment. Relapse was defined as a TB patient who was declared cured or treatment completed by a physician, but who reports back to the health service and is now found to be sputum culture positive. EDTA plasma samples were collected before initiation of ATT as well as at month 2 and 5 of treatment. Samples were frozen at −80°C until use in the immunoassays.

### Rabbit Lung Cavitation Model

The lung and plasma samples employed were from infected animals described in previously published papers (33, 34). In these studies, female New Zealand white rabbits, weighing 3–3.5 kg (Covance Research Products, Gaithersburg, MD, USA), were sensitized with five 0.2 ml subcutaneous injections of 1 × 10^7^ heat-killed *Mycobacterium bovis*, emulsified 1:1 in Freund’s adjuvant. Twenty-one days after sensitization, skin test reactivity was determined by injection of 5 IU of purified protein derivative (Tubersol; Sanofi-Aventis, Bridgewater, NJ, USA) and measured at 48 h. For infection, rabbits were anesthetized by intramuscular administration of ketamine (10 mg/kg) and xylazine (20 mg/kg) prior to endotracheal tube intubation. A 3.0-mm flexible Pentax FB-8V pediatric bronchoscope (Pentax, Montvale, NJ, USA) was guided into 1 lower lobe, and 400 µL of 7.5 × 10^3^ log-phase *Mtb* strain H37Rv was inoculated *via* catheter. At day 42 postinfection, EDTA plasma specimens were collected from the central ear artery. Rabbits were euthanized following anesthesia by intravenous injection of Euthasol (Virbac, Fort Worth, TX, USA). Different lung regions presenting with cavitation (cavity wall), granulomatous lesions, or normal lung tissue were dissected for protein analysis and snap frozen in liquid nitrogen as previously described (33, 34). In addition, 3 mm punch biopsies (six/tissue/animal) from each dissected lung region were weighed and then homogenized in phosphate-buffered saline (PBS) prior to *Mtb* CFU enumeration on 7H11 selective medium (33). The remaining lung lobe (>90% by mass) was weighed and then homogenized, using a Polytron homogenizer (Kinematica, CH), in 40 mL PBS prior to CFU enumeration by serial dilution on selective 7H11 medium.

### Murine Model of Pulmonary TB

C57BL/6J mice between 6 and 8 weeks of age were obtained from National Institute of Allergy and Infectious Diseases (NIAID) through a supply contract with Taconic Farms (Germantown, NY, USA). All mice were bred and housed at Association for the Assessment and Accreditation of Laboratory Animal Care accredited Biosafety level 2 and 3 facilities at the NIAID, NIH, following all the National Research Council Guide for the Care and Use of Laboratory Animals guidelines. All experiments using mice were approved by the NIAID Animal Care and Use Committee. Mice were infected with approximately 100 CFU of the H37Rv strain of *Mtb* using an aerosol chamber (Glas Col, Terre Haute, IN, USA).

To evaluate the effects of chemotherapy, mice infected for 28 days were treated with a cocktail containing the antibiotics rifampin (R) (10 mg/kg/mouse), isoniazid (H) (25 mg/kg/mouse), and pyrazinamide (Z) (150 mg/kg/mouse) (all from Sigma-Aldrich). After 60 days, treatment was changed to a cocktail consisting of RH alone. The drug stocks were prepared weekly and were orally administered by gavage 5 days per week. At the designated time points, mice were euthanized, blood was collected, and lungs were extracted and homogenized in PBS and stored at −80°C for later analysis.

### Inbred and Mutant Mouse Strains Used As Source of Macrophages in *In Vitro* Experiments

The donors were WT B6.SJL (CD45.1/1) mice or animals genetically deficient in the HO-1 transcription factor NRF-2 (NRF2^−/−^), myeloid differentiation primary response gene 88 (MyD88^−/−^), TIR-domain-containing adapter-inducing interferon-β (TRIF^−/−^), IL-10^−/−^, IFNAR^−/−^, FTH1^−/−^. Cells from p47phox^−/−^ or gp91phox^−/−^ (two distinct subunits of the NADPH oxidase system) mice, which lack a fully functional NADPH-oxidase system, were also used. MyD88^−/−^, TRIF^−/−^, IL10^−/−^, and IFNAR^−/−^ together with their respective WT controls were obtained from Taconic and p47phox^−/−^ (C57BL/6NTac background, Taconic) and gp91phox^−/−^ (C57BL/6J background, Jackson Lab) mice, were provided by Dr. Sharon Jackson (NIMHD, NIH). Bone marrow from FTH1^−/−^ and NRF2^−/−^ mice were provided by Dr. Miguel Soares (Instituto Gulbenkian de Ciências, Oeiras, Portugal). Bone marrow from miR-155KO mice were a gift from Dr. Stefan Muljo (NIAID, NIH). All mice were backcrossed for at least eight generations on a C57BL/6J or N background (indicated when exact genetic background was referred to original references or by investigator donating the animals for our use).

### Non-Human Primate Model of Pulmonary *Mtb* Infection

The plasma samples analyzed were from animals utilized in a previously published study (37). In brief, 10 cynomolgus macaques (*Macacca fascicularis*) were infected with *Mtb* (Erdman strain) *via* bronchoscope, using an inoculum of 40–500 CFU (Table S1 in Supplementary Material). When the monkeys developed active TB (between 54 and 173 days postinfection; see Table S1 in Supplementary Material), they were treated daily with orally delivered isoniazid (*N* = 4) (15 mg/kg/dose) or rifampin (*N* = 6) (20 mg/kg/dose). Prior to treatment initiation and after 2 months of treatment, each macaque was given a PET/CT scan, using ^18^F-2 fluoro-deoxyglucose (FDG) as a probe, and animals were matched for disease pattern in each treatment group. Change in FDG avidity in lungs over treatment was reported in Ref. (37). Plasma samples obtained from these animals were stored at −80°C prior to analysis.

### *In Vitro* Experiments

CD14^+^ column-purified human elutriated monocytes were obtained from peripheral blood of healthy donors from the NIH blood bank under Institutional Review Board-approved protocols of both the NIAID and the Department of Transfusion Medicine. Macrophages were generated by culturing monocytes in the presence of RPMI 1640 media containing 10% human AB serum and M-CSF 50 ng/mL (PeproTech, Rocky Hill, NJ, USA) for 7 days; fresh media with growth factor were added every 48 h, as previously described (71). This method of macrophage differentiation was chosen based on a recently published guideline (72). For infection experiments, cells were plated at a density of 10^6^ cells/well in 24-well plates in phenol and serum-free media (Opti-MEM; Life Technologies, Carlsbad, CA, USA).

Murine BMDMs were employed in a second group of experiments. Bone marrow cells were cultured in 30% L929 cell-conditioned medium for 7 days. An additional 10 mL of L929 cell-conditioned medium were added after 4 days of incubation. The resulting adherent macrophages were detached with cold PBS and seeded in 96-well plates at 10^5^ cells/well, containing serum and phenol-free medium at 37°C in 5% CO_2_.

Cells were exposed to WT H37Rv or an ESAT-6-deficient H37Rv mutant (a gift from Dr. Volker Briken, University of Maryland, College Park, MD, USA) *Mtb* strain at the indicated multiplicity of infection for 3 h, washed to remove extracellular bacteria, and cultured in serum-free media for 24 h in the presence or absence of the indicated concentrations of Tempol (4-hydroxy-Tempo; 4-hydroxy-2,2,6,6-tetramethylpi-peridine-*N*-oxyl, Sigma-Aldrich) or a TAT-conjugated NRF-2 peptide (TAT-14) that interacts with the Keap-1/NRF-2 complex (TOCRIS, Bristol, UK). In some experiments, recombinant ESAT-6 was delivered into the cytosol of infected macrophages using a fusion protein with the N-terminal fragment of the lethal factor of *B. anthracis* as previously described (10). The anthrax-protective Ag used in this procedure was also prepared as described previously (73). The two proteins at the indicated doses were added to cell cultures 3 h after *Mtb* infection and incubated for 24 h. Cobalt (III) pro-toporphyrin IX dichloride (CoPPIX, Frontiers Scientific, Logan, UT, USA) was used as a positive control to induce HMOX1 mRNA expression in macrophages (Figure S2 in Supplementary Material). Culture supernatants were then collected, sterile filtered, and stored at −80°C until use. Whole cell extracts were prepared following the manufacturer’s instructions for the HO-1 ELISA kit (Enzo Life Sciences). Nuclear extracts were obtained using a kit from Active Motif (Carlsbad, CA, USA).

### Immunologic Measurement of Cellular Products

Rabbit and mouse lungs were perfused with PBS and homogenized in PBS containing Complete Ultra protease inhibitor cocktail (Roche, Basel, Switzerland) and 2 mM phenylmethylsulfonyl fluoride (Sigma-Aldrich). Rabbit HO-1 protein expression levels were quantified in plasma and lung homogenates (after normalization of their protein concentrations) using an ELISA kit (CUSABIO, College Park, MD, USA). HO-1 levels were measured in serum and lung homogenates of mice by ELISA (ADI-960-071, Enzo Life Sciences, Farmingdale, NY, USA) and in EDTA plasma samples from NHP using a human ELISA kit (ADI-EKS-800, Enzo Life Sciences). The same kit was used to measure HO-1 levels in plasma samples from TB patients as well as in whole cell extracts of the monocyte-derived macrophages studied *in vitro*.

Protein levels of the transcription factor NRF-2 in nuclear extracts from mouse macrophages were measured by ELISA (CUSABIO). NRF-2 activation was determined in nuclear extracts using TransAM DNA-binding ELISA kits (Active Motif, Carlsbad, CA, USA). Lipid peroxidation in culture supernatants was quantified using a kit (Cayman Chemical, Ann Harbor, MI, USA), which measures the formation of MDA. DNA/RNA oxidation was quantitated in culture supernatants using an immunoassay from Cayman Chemical that detects all three oxidized guanine species: 8-hydroxy-2′-deoxyguanosine from DNA, 8-hydroxyguanosine from RNA, and 8-hydroxyguanine from either DNA or RNA. Results from this assay were expressed as the concentration of 8-OH-DG in each sample. Intracellular production of ROS in macrophage cultures was assessed by staining cells with the oxidative fluorescent dye probe, dihydroethidium (DHE) 5 mM (Invitrogen/Molecular Probes, Grand Island, NY, USA) for 30 min at 37°C in5% CO_2_, and then analyzed by flow cytometry, as described previously (11). The cells used for ROS assay had been previously detached from the culture plates using trypsin (0.25%), washed, and then re-suspended in phenol and serum-free medium. Results were plotted as the mean fluorescence intensity of each of the experimental groups. Extracellular superoxide production was quantified as previously described (74) by adding hydroxylamine (0.5 mM) during cell culture, which converts superoxide into nitrite plus nitrate, which were reduced by VCl_3_ treatment and quantified by Griess reaction (Sigma-Aldrich).

### *HMOX1* Gene Expression Assay

Total RNA was isolated from murine macrophages using the RNeasy Mini Kit, and residual DNA was digested using RNase-free DNase (both from QIAGEN, Valencia, CA, USA). The RNA samples were reverse transcribed using SuperScript II Reverse Transcriptase (Invitrogen, Carlsbad, CA, USA). Gene expression was measured using SYBR Green-based real-time quantitative PCR, and 18S mRNA was used as the housekeeping gene. The following oligonucleotide primers were used: 18S, forward, 5′-CACGGCCGGTACAGTGAAAC-3′ and reverse, 5′-CCCGTCGGCATGTATTAGCT-3′; and *HMOX1*, forward, 5′-TCTCAGGGGGTCAGGTC-3′ and reverse, 5′-GGAGCGGTGTCTGGGATG-3′. Fold induction of *HMOX1* gene expression was calculated using the DD threshold cycle method, normalizing mRNA levels for each sample to levels of 18S and comparing with mRNA levels in unstimulated cells.

### Data Analyses

The median values with interquartile ranges were used as measures of central tendency. For the *in vitro* experiments, bars represent mean and SD. The Mann–Whitney *U* test (for two groups) or the Kruskal–Wallis test with the Dunn’s multiple-comparison or non-parametric linear trend *post hoc* test (for more than two groups) were used to compare continuous variables in unmatched groups, whereas the Wilcoxon (for two groups) or Friedman (for more than two groups) matched-pairs tests were used in matched analysis, as indicated in table notes and figure legends. The Fisher’s exact test or chi-square was used to compare variables displayed as percentages. Spearman rank tests were used to assess correlations. ROC curves were used to test the power of HO-1 levels to distinguish cure (successful treatment) from treatment failure or TB relapse at week 20 of ATT. All comparisons were prespecified and two-tailed. Differences with *p* values below 0.05 after Holm’s adjustment for multiple comparisons were considered statistically significant. The statistical analyses were performed using GraphPad Prism 7.0 (GraphPad Software, La Jolla, CA, USA), STATA 11 (StataCorp, College Station, TX, USA), JMP 13.0 (SAS, Cary, NC, USA), and R 3.1.0 (R Foundation, Vienna, Austria) programs.

## Ethics Statement

The clinical study performed was approved by the Human Research Ethics Committee of the University of Cape Town, South Africa (approval number: 568/2012). Written informed consent was obtained from all study participants. The protocols, procedures, and animal care for the murine, rabbit, and non-human primate studies were approved, respectively, by the Institutional Animal Care and Use Committee from the National Institutes of Health (NIH), Bethesda, MD, USA (protocol number LPD99E); Johns Hopkins University, Baltimore, MD, USA (protocol number RB11M466); and University of Pittsburgh School of Medicine, Pittsburgh, PA, USA (protocol numbers A3187-01, 802011, and 808244). All the experiments were carried out in accordance with the recommendation of the Guide for the Care and Use of Laboratory Animals of the National Research Council of the National Academies of the United States of America (8th Edition) as mandated by the U.S. Public Health Service Policy. All animal experiments were performed in animal BSL-3 facilities maintained by the Intramural Research Program of National Institute of Allergy and Infectious Diseases (NIAID), Johns Hopkins University, and the University of Pittsburgh.

## Author Contributions

NR, DC, EA, BA, EB, AK, LG-S, KF, and CS performed experiments. NR, DC, EA, AS, RW, and BA designed experiments. AK, JF, SJ, KW, WB, AS, RW, and BA provided materials and infrastructural support. DC, EA, AS, RW, and BA wrote the manuscript.

## Disclaimer

The funders had no role in study design, data collection, and interpretation or the decision to submit the work for publication.

## Conflict of Interest Statement

The authors declare that the research was conducted in the absence of any commercial or financial relationships that could be construed as a potential conflict of interest.
