# Supplementary material for: Mycobacterium tuberculosis Induction of Heme Oxygenase-1 Expression Is Dependent on Oxidative Stress and Reflects Treatment Outcomes
Source: Front Immunol. 2017 May 12;8:542. doi: 10.3389/fimmu.2017.00542 (PMC5427075; doi:10.3389/fimmu.2017.00542)
Supplement: Supplementary file 1 [file data_sheet_1.pdf]

Supplementary Material

Mycobacterium tuberculosis Induction of Heme Oxygenase-1 Expression is Dependent on Oxidative Stress and Reflects Treatment Outcomes

Neesha Rockwood, Diego L. Costa, Eduardo P. Amaral, Elsa Du Bruyn, Andre Kubler, Leonardo Gil-Santana, Kiyoshi F. Fukutani, Charles A. Scanga, JoAnne L. Flynn, Sharon H. Jackson, Katalin A. Wilkinson, William R. Bishai, Alan Sher, Robert J. Wilkinson, Bruno B. Andrade

Corresponding authors:

Bruno B. Andrade
bruno.andrade@bahia.fiocruz.br (BBA)

Robert J. Wilkinson
r.j.wilkinson@imperial.ac.uk (RJW)

Supplementary Figures and Tables

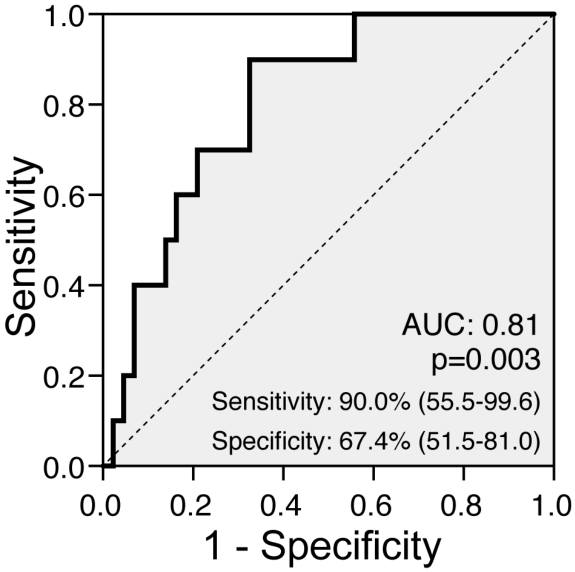

Fig S1. Discrimination of successful treatment from failure or relapse in TB patients at week 20 of antitubercular therapy.

Receiver Operator Characteristics (ROC) curve analysis of plasma HO-1 levels from TB patients at week 20 of antibubercular therapy was performed to test the performance of this marker in discriminating individuals who were successfully treated (negative sputum cultures and no symptoms) from those who underwent treatment failure or subsequently relapsed (see Methods for clinical details). HO-1 cut-off value used for the ROC inferential analysis was >11.5 ng/mL. AUC, area under the curve.

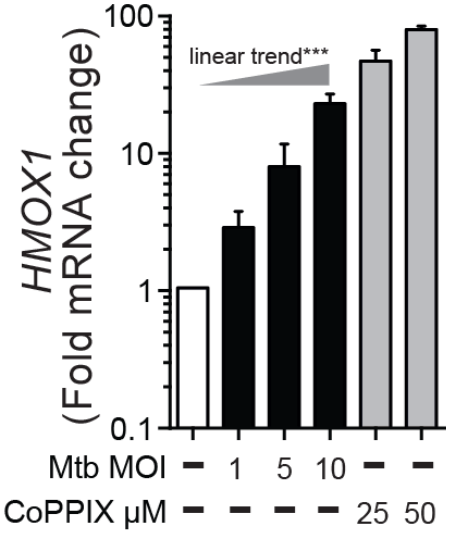

Fig S2. ***Mycobacterium tuberculosis*** **infection of murine bone marrow-derived macrophages induces** ***HMOX1*** **mRNA expression.** BMDM were infected with *M. tuberculosis* (Mtb) at indicated multiplicities of infection (MOI). *HMOX1* mRNA expression values (at 18h post infection) were plotted as fold change from unstimulated cultures as described in methods. Cultures were also treated for 18h with the HO-1 inducer cobalt protoporphyrin IX (CoPPIX) to serve as positive control for the expression assay (see Methods for details). Data are from at least three independent experiments using triplicate biological samples. Bars represent mean and SEM values. Data were compared using Kruskal-Wallis test with linear trend ad hoc test. ***p<0.0001.

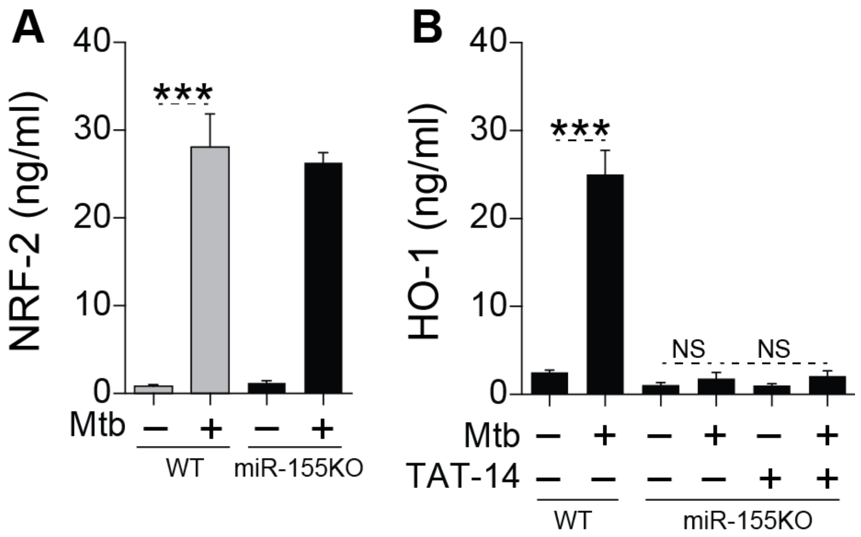

Fig S3. Bone marrow-derived macrophages from mice lacking miR-155 fail to induce HO-1 upon *M. tuberculosis* infection. BMDM from miR-155KO mice or their respective WT were infected with *M. tuberculosis* (MOI of 3). Protein expression of Nuclear factor erythroid-derived 2-like 2 (NRF-2) **(A)** and HO-1 **(B)** were quantified in nuclear extracts is 12h and whole cells lysates in 24h post infection, respectively by ELISA. In some conditions, cultures were treated with TAT-conjugated NRF-2 sequence peptide that interacts with the Keap-1/NRF-2 complex (TAT-14, 50µM). Bars and lines represent mean and SEM, respectively. Data are from at least three independent experiments using triplicate biological samples. Data were compared using the Wilcoxon matched-pairs test. ***p<0.0001, NS, nonsignificant.

Table S1. ***Mycobacterium tuberculosis*** **infection dose and time to treatment of cynomolgus macaques**

Monkey	Treatment	Infection dose (CFU)	Time to treatment (days post-infection)
19308	INH	40	73
8309	INH	310	54
9009	INH	225	152
5408	INH	486	155
5108	RIF	380	134
21608	RIF	40	66
7909	RIF	310	87
8909	RIF	190	173
4608	RIF	380	85
4708	RIF	486	45

INH, isoniazid; RIF, rifampicin.
